# Promising Drug Targets and Compounds with Anti-*Toxoplasma gondii* Activity

**DOI:** 10.3390/microorganisms9091960

**Published:** 2021-09-15

**Authors:** Marco da Silva, Cátia Teixeira, Paula Gomes, Margarida Borges

**Affiliations:** 1 Departamento de Ciências Biológicas, Faculdade de Farmácia da Universidade do Porto, 4050-313 Porto, Portugal; up201703294@edu.ff.up.pt; 2 LAQV-REQUIMTE, Departamento de Química e Bioquímica, Faculdade de Ciências da Universidade do Porto, 4169-007 Porto, Portugal; catia.teixeira@fc.up.pt (C.T.); pgomes@fc.up.pt (P.G.); 3 Associate Laboratory i4HB—Institute for Health and Bioeconomy, Faculty of Pharmacy, University of Porto, 4050-313 Porto, Portugal; 4 UCIBIO/REQUIMTE, Laboratory of Toxicology, Department of Biological Sciences, Faculty of Pharmacy, University of Porto, 4050-313 Porto, Portugal

**Keywords:** anti-*Toxoplasma* agents, drug discovery, drug repurposing, drug targets, *Toxoplasma gondii*, toxoplasmosis

## Abstract

Toxoplasmosis is a parasitic disease caused by the globally distributed protozoan parasite *Toxoplasma gondii*, which infects around one-third of the world population. This disease may result in serious complications for fetuses, newborns, and immunocompromised individuals. Current treatment options are old, limited, and possess toxic side effects. Long treatment durations are required since the current therapeutic system lacks efficiency against *T. gondii* tissue cysts, promoting the establishment of latent infection. This review highlights the most promising drug targets involved in anti-*T. gondii* drug discovery, including the mitochondrial electron transport chain, microneme secretion pathway, type II fatty acid synthesis, DNA synthesis and replication and, DNA expression as well as others. A description of some of the most promising compounds demonstrating antiparasitic activity, developed over the last decade through drug discovery and drug repurposing, is provided as a means of giving new perspectives for future research in this field.

## 1. Introduction

The parasite *Toxoplasma gondii* is an obligate intracellular protozoan that infects most warm-blooded animals [1]. However, its sexual lifecycle can only occur in members of the Felidae family, known as definitive hosts, such as domestic cats and wild felids, whose global *T. gondii* seroprevalence is estimated to be 35% and 59%, respectively [2]. The rates of *T. gondii* seroprevalence in humans vary greatly among different geographical areas, ranging from approximately 30% in the American, European, and Asiatic regions, to more than 60% in the African continent. These fluctuations may result from different cultural practices, environmental conditions, or the socioeconomic status of the population. Overall, *T. gondii* is considered one of the most successful parasites worldwide, infecting about 30% of the world’s population [3]. Despite its ubiquitous distribution, several divergent strains of *T. gondii* were detected.

Most strains isolated in Europe and North America fall into one of three genotypes, referred to as types I, II and III. Type I are considered the most virulent strains and are lethal in mice, displaying enhanced migratory capacity both in vitro and in vivo, the faster growth rate being in vitro, and being capable of generating high parasite loads in the host organism. Type II and III have intermediate and low virulence, respectively, with type II being the most common strains among human infections, while type III are mostly found in non-human infections. Certain strains are typically predominant in certain geographic locations. The three major clonal lineages are found in Asia and America, in addition to certain specific genotypes for the former area and various recombinant strains for the latter. Type II and III strains are isolated in North Africa, the Middle East, and the Arabic peninsula, while in Europe, type II strains are more common. Several African genotypes, including type II and III strains, are found in sub-Saharan Africa [4,5,6].

## 2. Toxoplasmosis

In humans, the severity of infection is mainly determined by the host immune system efficacy. In immunocompetent individuals, acute *T. gondii* infection is usually asymptomatic or may cause, in rare instances, a flu-like disease with mild symptoms. Strain virulence and several host-cell conditions—pH, heat shock, mitochondrial inhibition, and nitric oxide production—may also influence disease outcome [4,7,8]. Under host immune pressure, the parasite naturally becomes dormant. In this process, tachyzoite–bradyzoite conversion occurs, with tissue cyst formation in specific locations, such as cerebral, skeletal, and cardiac muscle tissues, reaching the chronic phase of infection. An intact immune system prevents cyst rupture and reactivation of infection [4,7]. However, in immunocompromised individuals, the parasite generally remains active, causing continuous host-cell infection, leading to acute disease. Moreover, reactivation may also occur in individuals who are initially immunocompetent but later undergo immunosuppression, including individuals with acquired immunodeficiency syndrome (AIDS), or subjected to immunosuppressive therapy, as in the case of autoimmune diseases or organ transplantation. Individuals with AIDS and not initially infected with *T. gondii* usually develop a severe primary infection, whereas individuals already infected with the parasite who become immunosuppressed are at an increased risk of disease relapse [9,10]. In fact, *T. gondii* encephalitis was frequently reported in AIDS patients, especially those with low CD4 T lymphocyte cell counts. Therefore, *T. gondii* may be regarded as an opportunistic parasite that contributes to the death of AIDS patients [10]. In these situations, where *T. gondii* infection is acquired throughout an individual’s life, the disease is referred to as acquired toxoplasmosis.

Toxoplasmosis may also be potentially dangerous in seronegative pregnant women that become primo-infected during pregnancy, as it may lead to transplacental transmission of the parasite, which may result in congenital toxoplasmosis [11]. However, the incidence of congenital toxoplasmosis varies with the trimester during which primary infection is acquired. The transmission rate is greater in the final stages of pregnancy, as placental irrigation increases, allowing for a greater area of contact with the fetus. On the other hand, the severity of infection is greater in the early stages of pregnancy, due to fetal immunological immaturity [12,13,14]. During the first trimester, there is a higher risk of abortion, stillbirth, or premature birth. In the second trimester, the risk of miscarriage decreases, however, in more severe cases, hydrocephaly, chorioretinitis and cerebral calcification may occur, according to the parasite’s brain and ocular tropism. In the last trimester, although severe clinical manifestations in the newborn are at lower risk, there is an increased probability of congenital infection [11,15,16,17,18].

Regarding cerebral tropism, recent data suggest an association between congenital infection and the development of neurological and psychiatric disorders later in life [9,19,20], including Alzheimer’s disease [19,20], depression [19,20,21], schizophrenia [22,23,24], bipolar disease [24], and even suicidal tendencies [21,24].

## 3. Current Treatment Options

First-line conventional treatment for acquired and congenital toxoplasmosis generally includes a pyrimethamine-based regimen, which comprises three drugs: pyrimethamine, sulfadiazine and folinic acid (leucovorin; Table 1) [25,26]. Pyrimethamine is a folic acid antagonist as it inhibits the dihydrofolate reductase (DHFR) enzyme, blocking the synthesis of purines and pyrimidines, essential for DNA synthesis and cell multiplication. The action of this drug is enhanced when used in conjunction with sulfonamides, such as sulfadiazine, which is capable of interfering with *T. gondii*’s folic acid synthesis, by competitively inhibiting the dihydropteroate synthetase (DHPS) enzyme. This combination must not be used during the first trimester of pregnancy due to the teratogenic potential of pyrimethamine, which also causes reversible myelosuppression, forcing combination with folinic acid, to prevent hematologic adverse reactions [25,26,27,28]. Moreover, although rare, different severe complications were reported, such as agranulocytosis, Stevens-Johnson syndrome, toxic epidermal necrolysis and hepatic necrosis, as well as many others [29,30,31,32,33,34]. Although several alternative treatment options are available, including pyrimethamine combined with clindamycin, clarithromycin, azithromycin or atovaquone, and monotherapy using cotrimoxazole (trimethoprim-sulfamethoxazole) or atovaquone, no regimen was found to be more effective than the conventional treatment [25,35]. Despite clinical complications, standard chemotherapy has proven to reduce the risk of development of toxoplasmosis-related sequels and symptoms associated with congenital infection in newborns, if it is administered immediately after diagnosis of either maternal infection or congenital transmission [25,27,36]. Alternatively, when the maternal infection is suspected, but not confirmed, therapy with spiramycin must be implemented. Spiramycin is a potent macrolide antibiotic, and although it does not readily cross the placental barrier, it is greatly accumulated in the placenta, preventing transplacental transmission of *T. gondii*. Nevertheless, when fetal or neonatal toxoplasmosis is confirmed, spiramycin is discontinued and conventional treatment is applied [36]. The use of steroids is beneficial in the treatment of ocular toxoplasmosis in combination with antimicrobial therapy. However, excessive doses can lead to a minimal response [25]. In fact, phase II clinical trials are currently underway to determine the optimal dose of dexamethasone to be used as adjunctive therapy to reduce brain edema in HIV-infected patients exhibiting cerebral toxoplasmosis [37].

Current treatment options are limited and not optimal regarding the harsh profile of side effects and treatment duration (from 4–6 weeks to over 1 year), which may affect compliance [27,38]. *T. gondii*-related factors, such as the increasing drug resistance, different drug susceptibility for different strains, and the remaining unknown aspects of the parasite’s pathogenicity, also play an important role in disease progression and treatment failure [39,40,41]. In addition, no current drug can eliminate tissue cysts from the infected host, which remain quiescent, establishing the latent phase of infection, as long as the host’s immune system remains capable enough [27,38]. Although immunization strategies are currently being studied and developed, there is no vaccine available for human administration [42].

Thus, there is an urging need for the development of newer, safer, and more effective treatment alternatives for toxoplasmosis, which consequently relies on rising knowledge in *T. gondii* pathophysiology and the discovery of promising drug targets.

This review highlights some of the most promising drug targets in anti-*T. gondii* drug discovery and the compounds discovered, developed, and repurposed over the last decade, while focusing on their relevant features, in vitro and in vivo activity, and future perspectives. A literature search was conducted using PubMed database and the query “((toxoplasmosis) OR (*Toxoplasma gondii)* OR (anti-*Toxoplasma*) AND (drug) OR (treatment)). Experimental compounds with established in vitro activity were primarily considered, of which those with in vivo activity or efficacy against bradyzoite-containing cysts were prioritized.

## 4. Promising Drug Targets and Strategies in Anti-*T. gondii* Drug Discovery

**Figure 1:**
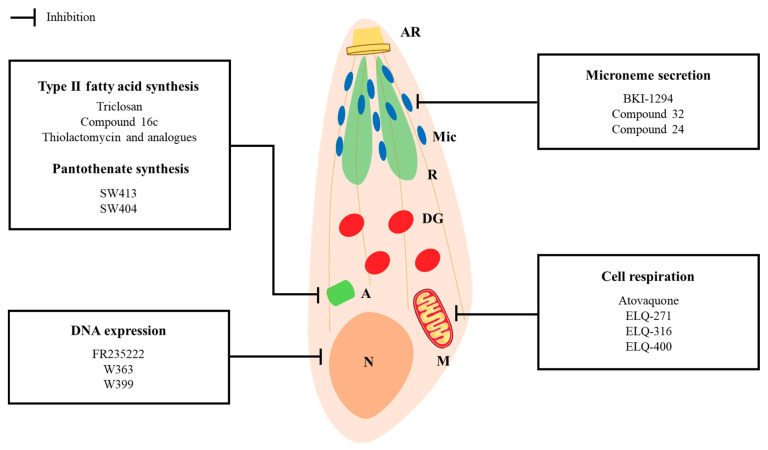
Graphical representation of a *Toxoplasma gondii* tachyzoite. Organelle and pathway targets of several experimental and repurposed compounds [43,44,45,46,47,48,49,50,51,52,53,54,55,56]. AR: apical ring; Mic: microneme; R: rhoptry; DG: dense granule; A: apicoplast; M: mitochondrion, N: nucleus; BKI: bumped-kinase inhibitor; ELQ: endochin-like quinolone.

In the last decade, considerable efforts have been made in the study and development of repurposed drugs and novel compounds with new mechanisms of action. Drug screens involved various parasite targets, including mitochondrial electron transport chain, calcium-dependent protein kinases, type II fatty acid synthesis, DNA synthesis and replication, and DNA expression as well as many others. Drug targets and respective promising inhibitors with interesting mechanisms of action (Figure 1) and efficient in vitro and/or in vivo activity against *T. gondii* are described and discussed. Relevant experimental in vitro and in vivo results are summarized in Table 2.

### 4.1. Drug Targets Involved in Parasite Motility and Host-Cell Invasion

**Figure 2:**
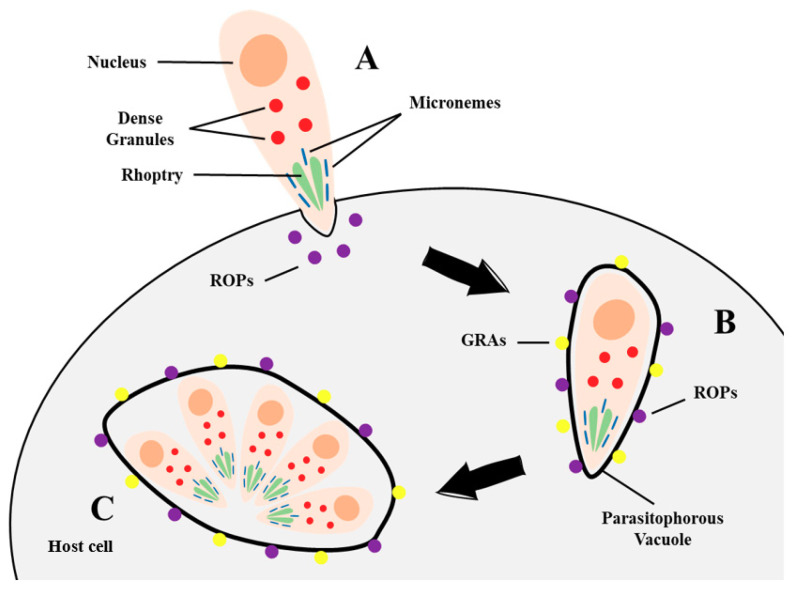
*Toxoplasma gondii* host-cell invasion and establishment of intracellular parasitic compartments [68,69,70,71,72]. (**A**) To invade the host cell, the parasite requires the secretion of proteins from two organelles, micronemes and rhoptries. These proteins allow the parasite to enter the host cell while coating itself with the host cytoplasmatic membrane, forming the Parasitophorous Vacuole (PV). (**B**) Upon entry, the PV is decorated with rhoptry proteins (ROPs) and proteins derived from the third set of organelles, known as dense granules (GRAs). (**C**) When the parasite is established inside the PV, it replicates asexually, generating a large enough number of tachyzoites capable of rupturing the host cell and infecting surrounding cells or, if in specific tissues or under certain conditions, convert into metabolically less competent bradyzoites and form tissue cysts.

*T. gondii* belongs to the Apicomplexa family, which also includes other relevant protozoans, such as *Cryptosporidium* spp., a common cause of diarrhea in children, and *Plasmodium* spp., the etiological agent of malaria. In fact, both *T. gondii* and *Plasmodium* spp. share very similar organellar organization [67]. The members of this family possess well-developed structures at the anterior end of the cell—the apical complex—responsible for host-cell invasion [68]. Unlike *Plasmodium* spp., which specifically infects erythrocytes and hepatocytes, *T. gondii* does not require a specific host receptor for cell invasion, allowing it to infect all nucleated host cells [67]. The invasion process (Figure 2) requires the participation of specific *T. gondii* secretory organelles, belonging to the apical complex: the micronemes, small rod-shaped structures accumulated in the apical third of the protozoan body, housing proteins responsible for extracellular motility and invasion; and rhoptries, long club-shaped organelles located at the apical portion of the parasite, which accommodate proteins responsible for the invasion and host-cell manipulation. Proteins segregated by micronemes (named MICs) and rhoptries (named ROPs) allow host-cell entrance while dragging cytoplasmatic membrane around the tachyzoite, forming the Parasitophorous Vacuole (PV)—an intracellular compartment in which *T. gondii* reproduces asexually. Upon the formation of the PV, the third set of proteins derived from other secretory organelles, the dense granules (GRAs), along with ROPs, decorate the PV membrane [68,69,70,71]. Other ROPs and GRAs accumulate inside PV forming a tubular network of intravacuolar structures that serves various purposes: escape from host-cell aggression, inhibit phagolysosome formation, hinder intravacuolar acidification, metabolic exchange of compounds between PV and host cytoplasm, among others [69,72]. Interaction between *T. gondii* and host cell endocytic machinery is well described, however, the infection may occur through other mechanisms, such as the chlathrin-mediated endocytosis or micropinocytosis [73]. These processes are essentially controlled by the host cell, revealing a successful interplay between host cell and parasite in *T. gondii* infection [70].

#### *T. gondii* Calcium-Dependent Protein Kinase 1

MICs secretion is essential for parasite motility, host cell invasion, and egress and thus constitutes a potential target for drug development. In fact, the inhibition of *T. gondii* calcium-dependent protein kinase 1 (*Tg*CDPK1), a member of the serine/threonine-protein kinase family located in the cytosol and regulates the calcium-dependent pathway, which in turn leads to MICs secretion, impairs host-cell invasion capacity [74,75]. *Tg*CDPK1 has thus proven to be an interesting target for drug discovery. In comparison to mammalian kinases, it presents a key structural difference at the “gatekeeper residue” in the ATP-binding pocket. *Tg*CDPK1 contains a small glycine residue, whereas human kinases possess larger residues, providing additional space for extra interactions with the target protein, which resulted in the development of potent and selective ATP-competitive *Tg*CDPK1 inhibitors [75,76,77].

Several bumped kinase inhibitors (BKI) were found to selectively inhibit *Tg*CDPK1, being 15,000-fold more active against the parasite kinase in comparison to human tyrosine kinases [43,78]. BKI-1294, a pyrazolo-pyrimidine based compound, was a promising candidate belonging to this class. Doggett et al. described an in vitro IC_50_ of 140 nM, leading to a reduction in acute *T. gondii* infection by 93% when given orally, and high efficiency against established toxoplasmosis [43]. In addition, Müller et al. demonstrated excellent activity of BKI-1294 against congenital toxoplasmosis [57]. However, despite elevated *T. gondii* specificity, BKI-1294 was found to inhibit the *Ether-à-go-go-Related Gene* (*hERG*), which codes for the protein Kv11.1, the alpha subunit of a potassium ion channel that is essential in cardiomyocyte repolarization. Its inhibition may ultimately result in the development of life-threatening cardiac arrhythmias, such as *torsades de pointes* [44,58,79]. This occurrence halted BKI-1294 development due to the risk of cardiotoxicity. Nevertheless, Sánchez-Sánchez et al. recently demonstrated the safety and significant protection provided by BKI-1294 against abortion and vertical transmission in sheep experimentally infected with *T. gondii* during pregnancy. Thus, although BKI-1294 advancement for human toxoplasmosis ceased, the reduction of infection rates among other intermediate hosts may be a way to indirectly reduce human infection [59].

Vidadala et al. investigated BKI-1294 modifications that maintained *Tg*CDPK1 selectivity and efficacy while reducing interference with hERG channels. The authors developed a compound (compound 32 in their series of BKIs) with an hERG IC_50_ > 10 µM, an in vitro IC_50_ against *T. gondii* of 60 nM, and high in vivo efficiency regarding brain parasite load when given orally. In fact, compound 32 reduced the number of brain cysts by 88.7% [44].

Recently, Rutaganira et al. tested other pyrazolo-pyrimidine inhibitors of TgCDPK1. The resulting compound (compound 24 in their series of BKIs) exhibited in vitro inhibition of the enzyme and parasite proliferation in the nanomolar and submicromolar range, respectively. In addition, in vivo assays showed this BKI analog to exhibit excellent oral bioavailability, decreased severity of acute infection, reduced cyst burden and delayed chronic reactivation of disease in immunocompromised mice. Noteworthy, compound 24 was able to completely cure some of the immunocompromised animals [45].

### 4.2. Drug Targets Involved in Fatty Acid Synthesis

The fatty acid synthesis (FAS) pathway can provide attractive approaches in *T. gondii* drug development, especially since several drug targets include enzymes absent in the host cell. As with other metabolic pathways, FAS takes place in the apicoplast of apicomplexan parasites [80,81]. Whilst *T. gondii* can effectively scavenge host cell precursors, the fatty acids produced in the apicoplast are essential for parasite development and survival [81,82]. In fact, the parasite can sense lipid availability in the surrounding environment, allowing a proper balance between *de novo* synthesis and nutrient scavenging pathways, to maintain membrane genesis, which is important for division, growth and overall survival and pathogenesis [83]. Elongation of nascent fatty acids in the FAS II pathway is a process mediated by multiple enzymes located in the endoplasmic reticulum, which are also present in bacteria and plants. In animals and fungi, the elongation process is catalyzed by the type I FAS pathway, leading to a single large multifunctional polypeptide [80,83,84]. Since lipid synthesis is regarded as an essential process, especially during tachyzoite intracellular development, for membrane genesis and lipid homeostasis and signaling, its inhibition is of great interest in anti-*T. gondii* drug discovery [83].

#### 4.2.1. The FAS II Enzyme Enoyl-Acetyl Carrier Protein Reductase (ENR)

The FAS II apicoplast-located enzyme enoyl-acetyl carrier protein reductase (ENR) catalyzes the last step in fatty acid synthesis [46,82]. ENR is inhibited by triclosan, an antibacterial compound, which inhibits *T. gondii* in vitro growth at low micromolar to nanomolar concentrations [46]. However, triclosan has poor solubility and oral bioavailability. El-Zawawy et al. described a liposomal-based delivery system for triclosan, which was able to reduce, in vivo, both tachyzoite and cyst burden. In fact, the incorporation of triclosan within the liposomes allowed the use of lower dosages, undermining possible adverse effects, whilst maintaining its antiparasitic activity [47,60]. Stec et al. later reported several promising triclosan analogs with better anti-*Toxoplasma* activity than the parent compound, such as compound 16c, of this series, presenting an in vitro IC_50_ of 250 nM, compared to 3 µM of triclosan. In addition, an overall improvement in pharmacokinetics was also observed. However, despite promising in vitro results, in vivo assays only showed decreased *T. gondii* proliferation in mice at much higher doses (75 mg/kg) when compared to the most effective doses used in triclosan assays (10 mg/kg). Nevertheless, compound 16c was 10–fold less toxic than triclosan in vivo [61]. Overall, the promising in vitro activity and interesting pharmacokinetic profile of compound 16c makes it a potential scaffold for further development.

#### 4.2.2. β-Ketoacyl-Acyl Carrier Protein Synthase I and II (KAS I/II)

The β-ketoacyl-acyl carrier protein synthases I and II (KAS I/II) are other drug targets belonging to the FAS II pathway and that are essential for fatty acid elongation. In fact, mutants that lack these enzymes are deficient in unsaturated fatty acids [48]. KAS I/II are specifically inhibited by thiolactomycin and its analogs. Martins-Duarte et al. determined IC_50_ values between 1.6 and 29.4 µM, and electron microscopy studies of treated parasites revealed serious morphological alterations in parasite shape and intracellular organelle organization. Treatment with these compounds resulted in swollen mitochondria with disrupted structures, enlarged Golgi complex, and expanded endoplasmic reticulum throughout the whole parasite cytoplasm. The replication process (endodyogeny) was also affected, as uncompleted division processes were observed, resulting in large multinucleated parasites. These findings are indicative of parasite toxicity and death, so thiolactomycin and analogs show a clear impact in parasite development and survival [62]. However, to the best of our knowledge, in vivo testing of these compounds has not yet been reported.

#### 4.2.3. Pantothenate Synthetase

*T. gondii* is capable of synthesizing FAS precursors outside the FAS II pathway, such as pantothenate, a coenzyme A precursor. The parasite’s pantothenate pathway includes the terminal enzyme pantothenate synthetase that converts pantoate to pantothenate. Unlike humans, *T. gondii* does not require an external source of pantothenate [49]. Host cell conversion of pantothenate to coenzyme A is rather rapid, preventing *T. gondii* from scavenging the precursor from the host cell [27]. Thus, de novo pantothenic acid synthesis can be an attractive target for drug discovery, as it avoids interference with host cell FAS. Mageed et al. tested several acylsulfonamides, originally developed to target *Mycobacterium tuberculosis* pantothenate synthetase. In order to assess parasite growth inhibition, the commonly used drug pyrimethamine served as a positive control. Compounds SW413 and SW404 of this series demonstrated in vitro IC_50_ values of 20 and 130 nM, respectively, with median toxic doses (TD_50_) above 1000 µM in human foreskin fibroblasts (HFF). These inhibitors were at least as potent as pyrimethamine regarding parasite growth inhibition. To infer whether pantothenate synthetase is the possible target of the compounds, the EC_50_ values were assessed in the presence and absence of supplemental pantothenate. The addition of pantothenate increased the EC_50_ values of both SW413 and SW404, indicating that *de novo* pantothenate synthesis is essential for *T. gondii* survival and can be effectively targeted [49]. Regardless, in vivo experiments are needed to examine the efficacy of these candidates in animals.

### 4.3. Drug Targets Involved in DNA Expression

*T. gondii* is capable of rapidly differentiating between the active tachyzoite stage and the slow-growing bradyzoite stage. This tachyzoite–bradyzoite interconversion process requires the expression and modulation of stage-specific genes. This modulation may be performed through epigenetic mechanisms, using a post-translational modification (PTM) of histone proteins. PTMs include acetylation or deacetylation of histone residues [85,86]. Acetylation of conserved histone lysine residues by histone acetyltransferases (HATs) generates PTMs that generally lead to increased target gene expression. Instead, deacetylation of these residues by histone deacetylases (HDACs) removes the modification, resulting in decreased target gene expression [50,51]. HATs and HDACs that target stage-specific genes contribute greatly to parasite interconversion between tachyzoite and bradyzoite stages. Modulation of gene expression in this regard may contribute greatly towards the development of treatment options for chronic toxoplasmosis. Preventing bradyzoite differentiation may help to avoid chronic infection, whereas preventing tachyzoite conversion may avoid prompt reactivation of *T. gondii* acute infection in immunocompromised patients [38]. In addition, this PTM is also present in other drug targets, including several proteins involved in DNA repair, including the chaperone Hsp90 and the ATM serine/threonine-protein kinase [87]. Therefore, epigenetic regulation of gene expression can be considered an attractive idea for *T. gondii* drug development.

#### Histone Deacetylase Enzyme TgHDAC3

Histone deacetylase enzyme TgHDAC3 proved to be an effective drug target for *T. gondii* inhibition [52]. The cyclopeptide FR235222 targets TgHDAC3, causing hyper-acetylation of histone H4 in *T. gondii*. The compound demonstrated in vitro inhibition of intracellular parasite growth with an IC_50_ of 9.7 nM, induced in vitro bradyzoite conversion, and was able to reach bradyzoites within ex vivo cysts, preventing tachyzoite conversion. This promising result was further confirmed as FR235222-pretreated bradyzoites were found to be incapable of infecting HFF monolayers, and unable to cause toxoplasmosis in the mouse model. However, host cell toxicity was observed in FR235222 treatment, causing HFF cell inhibition at an IC_50_ of 128 nM. Further development of FR235222 analogs led to W363 and W399, which demonstrated higher parasite selectivity in comparison to the parent compound while maintaining equivalent parasite IC_50_ values in vitro [53]. The efficacy of these three compounds in chronically infected mice remains to be described.

### 4.4. Drug Targets Involved in Mitochondrial Electron Transport Pathway

#### Mitochondrial Cytochrome bc1 Complex

The cytochrome bc1 complex (bc1), present in the mitochondrial electron transport chain, is a drug target for several apicomplexan parasitic infections, including toxoplasmosis, malaria and babesiosis. Mitochondrial bc1 inhibitors bind to the hydroquinone oxidation (Qo) or quinone reduction (Qi) site of this complex, hindering cell respiration by inhibition of the electron transport pathway [27]. Atovaquone, which is clinically available for alternate treatment and prophylaxis of toxoplasmosis, as well as malaria and babesiosis, is a well-described Qo-site inhibitor. However, due to mutations in the target binding site, the development of resistance limited its use in toxoplasmosis [88].

Endochin-like quinolones (ELQ), which are 4-(1H)-quinolone derivatives, target the Qi site of bc1. The most promising compounds, ELQ-271 and ELQ-316, effectively exhibited low IC_50_ values for parasite growth inhibition, such as 0.100 and 0.007 nM, respectively. ELQ-271 inhibited human bc1 at 800 nM and ELQ-316 did not show human bc1 inhibition and was not toxic to HFF or human hepatocarcinoma cells (HepG2) at the highest concentration tested (10 µM). A reduction in the number of brain cysts by 88% in mice treated five weeks after infection with an ME49 *T. gondii* strain (type II strain) was also verified [54,55,56,63]. Due to the broad anti-apicomplexan activity of ELQ-316, combination with atovaquone was suggested to overcome the resistance issues associated with the latter [27]. Recently, McConnell et al. identified the compound ELQ-400 (also known as MMV671636, from *Neospora caninum* assays) as a promising candidate, capable of inhibiting both Qo and Qi sites of bc1 due to its structural flexibility and favorable substitution pattern. ELQ-400 decreased acute infection with a lethal type I strain of *T. gondii* in mice. In this assay, all mice survived and presented no signs of infection. ELQ-400 and ELQ-316 were simultaneously evaluated in vivo. Results indicate that both compounds are remarkably effective in decreasing infection in mice, however, they differ in tissue distribution and ability to prevent *T. gondii* from accessing the brain tissue, due to their distinct blood-brain barrier penetration capacity and half-life. In this regard, ELQ-400 was more effective in preventing the parasite from reaching the brain. Therefore, this compound is thought to effectively act on both acute and chronic phases of *T. gondii* infection [56,64].

## 5. Drug Repurposing Approach

Drug repurposing is a strategy for identifying new clinical uses for existing drugs with specific therapeutic indications [89]. This process is becoming an interesting strategy for drug discovery, as it involves potentially lower financial costs in drug development as well as shorter timelines [90].

The Medicines for Malaria Venture (MMV) foundation aims to reduce the burden of malaria by developing and facilitating access to new drug candidates. Thus, an open-access compound library called Malaria Box was made available for academics worldwide, with the purpose of identifying novel bioactive compounds against various pathogens. This library contained 400 blood-stage active anti-*Plasmodium* compounds. The screening led to the identification of seven potent anti-*T. gondii* compounds. Among these, the most potent and selective was the piperazine acetamide MMV007791 (compounds provided by the MMV foundation are generally designated by their MMV identifier codes) [91]. In 2015, MMV launched a novel open-access library, modeled after the Malaria Box, known as the Pathogen Box. It consists of 400 drug-like small molecules, contained in 96-well plates, with confirmed bioactivity against at least one of the following pathogens: *Plasmodium*, *Mycobacterium*, Kinetoplastids (such as *Trypanosoma*), *Schistosoma*, *Cryptosporidium*, helminths, and dengue virus. Eventually, since *T. gondii* is an apicomplexan parasite and morphologically similar to some of the parasites contained in this list, many compounds were found to have activity against this protozoan and even shed new insights into its biological pathways [64,65,92,93]. Spalenka et al. screened all 400 compounds for anti-*T. gondii* activity. Fifteen compounds demonstrated desired efficacy, of which eight presented selectivity and favorable in vitro effects on tachyzoite proliferation. The most active compound, MMV675968, is a diaminoquinazoline with known activity against the enzyme DHFR of *Cryptosporidium*, potentially targeting the same enzyme in *T. gondii*. This hypothesis was later assessed, and the compound exhibited an IC_50_ of 0.02 µM and high selectivity towards parasite DHFR enzyme. Buparvaquone (MMV689480), a well-known hydroxynaphthoquinone with described activity against *N. caninum*, by inhibiting several enzymes involved in the mitochondrial electron transport pathway, was also identified as having good in vitro anti-*T. gondii* activity, with an IC_50_ of 0.10 µM [65].

Recently, Murata et al. screened a chemical compound library, provided by the Drug Discovery Initiative from the University of Tokyo, and two promising compounds with anti-*T. gondii* activity were identified: tanshinone IIA, a compound with potential cancer cell growth inhibition; and hydroxyzine, a well-known first-generation antihistamine drug. *T. gondii* targets and mode of action are currently not known for these compounds, however, they were identified as effective in vitro inhibitors of tachyzoite growth, with reduced host cell toxicity. Moreover, tanshinone IIA and hydroxyzine showed inhibitory effects on the growth of bradyzoites. Thus, data indicate that these compounds may represent novel lead compounds to treat acute toxoplasmosis as well as preventing reactivation of latent infection, particularly in immunocompromised individuals [66]. However, in vivo efficacy remains to be reported.

## 6. Concluding Remarks

Toxoplasmosis is widely distributed worldwide, and the current chemotherapy lacks efficacy and safety. Clinically available options are associated with a relevant spectrum of adverse side effects and generally induce poor compliance within patients. Drug discovery has developed enormously in the last decade, with several drug candidates showing promising results both in vitro and in vivo. This review underlines several promising compounds, drug targets and strategies for anti-*T. gondii* drug development. Molecular modifications play an important role in this regard, with the aim of improving pharmacokinetic characteristics, including blood–brain barrier access, bioavailability, and half-life. The use of liposomal nanoparticles may also be applied in drugs with promising in vitro results that lack the necessary pharmacokinetic profile. Epigenetics and modulation of gene expression offer vast possibilities in drug discovery, as *T. gondii* provides a unique HAT/HDAC system that allows various explorative strategies. Drug repurposing has been used for several decades in many different diseases and should be continuously explored.

It is of great importance to invest in the study of novel potent drug candidates. Compounds exhibiting fewer and milder side effects, being overall better tolerated in pregnant women and newborns, specifically, in comparison to current chemotherapy, should be prioritized. Ideally, candidates should be capable of acting on both acute replicating tachyzoite and latent bradyzoite stages, preventing acute disease and reactivation, and even allowing resolution of chronic infection. In addition, the drugs should be bioavailable, capable of reaching therapeutic concentration in all target tissues, such as the brain and eye, as well as concentrate appropriately in the placenta and fetal compartment, while avoiding the generation of drug-resistant strains. Finally, drug costs should be affordable, providing treatment in all world regions. The development of such drugs would revolutionize current *T. gondii* chemotherapy.

## Figures and Tables

**Table 1:** Current drugs used for toxoplasmosis treatment.

Compound	Chemical Structure *	Mechanism of Action	References
Pyrimethamine	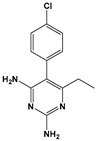	Antifolate	[25,27]
Sulfadiazine	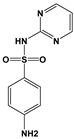	Antifolate	[25,27]
Folinic acid	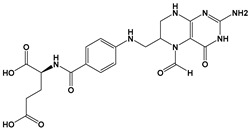	Reduction of pyrimethamine side effects	[25,27]
Spiramycin	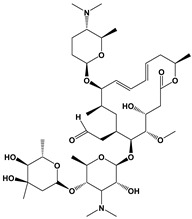	Protein synthesis inhibitor	[25,35]
Clindamycin	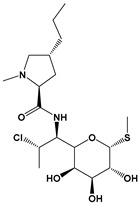	Protein synthesis inhibitor	[25,35]
Clarithromycin	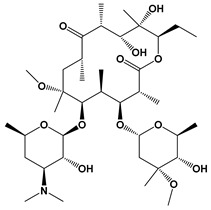	Protein synthesis inhibitor	[25,35]
Azithromycin	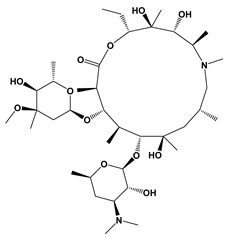	Protein synthesis inhibitor	[25,35]
Atovaquone	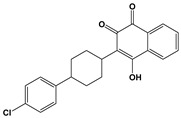	Mitochondrial electron transport chain inhibitor	[25,27]
Cotrimoxazole	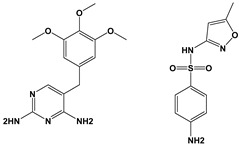	Antifolate	[25,27]

* Chemical structures were designed using ChemDraw 17.0 software (PerkinElmer Informatics, Waltham, United States of America).

**Table 2:** Experimental compounds with anti-*Toxoplasma gondii* activity.

Compound	Chemical Structure *	Drug Target	Affected *T. gondii* Pathway	In vitro IC_50_/*T. gondii* Strain/Host Cell	In vivo Results/*T. gondii* Strain/Animal Model/Infection Route	References
BKI-1294	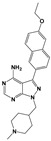	TgCDPK1	Parasite microneme secretion	140 nM/ RH/ HFF	93% reduction of parasite burden at 30 mg/kg. No *T. gondii* detected in peritoneal fluid of half the mice at 100 mg/kg/ RH/ CF-1 mice/ Intraperitoneal Protection against abortion and vertical transmission in sheep experimentally infected with *T. gondii* tachyzoites during pregnancy	[43,44,57,58,59]
Compound 32 ^a^	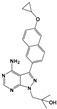	TgCDPK1	Parasite microneme secretion	60 nM/ ME49/ HFF	88.7% reduction in the number of brain cysts/ ME49/ CBA/J mice/ Oral gavage	[44]
Compound 24 ^a^	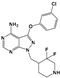	TgCDPK1	Parasite microneme secretion	TgCDPK1 inhibition at 10.9 nM (enzyme activity assay) Inhibition of parasite proliferation at 0.264 µM/ RH/ HFF	Decreased severity of acute infection. Delayed chronic reactivation of disease. Completely cured part of the animals/ ME49/ BALB/c mice and mice lacking IFN-γ receptor/ Oral gavage	[45]
Triclosan	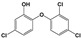	ENR	FAS II	3 µM/ RH/ HFF	Reduction in mice mortality, parasite burden and viability. Poor solubility and oral bioavailability/ RH/ Swiss albino mice/ Intraperitoneal	[46,47,60]
Triclosan-liposomal	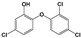 Liposomal	ENR	FAS II	ND	Reduction in host mortality and *T. gondii* brain burden by 98%/ ME49/ Swiss albino mice/ Oral gavage	[46,47]
Compound 16c ^b^	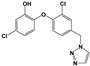	ENR	FAS II	250 nM/ RH/ HFF	Improvement in pharmacokinetics in comparison to triclosan Decreased peritoneal burden of *T. gondii*/ RH/ Swiss albino mice/ Intraperitoneal	[61]
Thiolactomycin (and analogs)	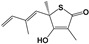	KAS I/II	FAS II	1.6–29.4 µM/ RH/ LLCMK2 Serious morphological alterations in treated parasites (electron microscopy)	ND	[62]
SW413 ^c^	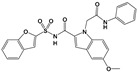	Pantothenate synthetase	Fatty acid chain elongation (within FAS II)	20 nM/ RH/ HFF	ND	[49]
SW404 ^c^	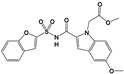	Pantothenate synthetase	Fatty acid chain elongation (within FAS II)	130 nM/ RH/ HFF	ND	[49]
FR235222	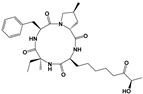	TgHDAC3	DNA expression	9.7 nM// RH/ HFF Dramatic morphological alterations in in vitro-induced bradyzoites at 30 nM Prevented bradyzoite-tachyzoite conversion (*ex vivo* cysts) at 200 nM	Reduced tachyzoite infection in mice. No brain cysts detected. No detectable humoral response/ PRU/ Outbred female Swiss mice/ Intraperitoneal	[53]
W363 ^d^	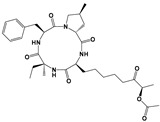	TgHDAC3	DNA expression	10.2 nM/ RH/ HFF	ND	[53]
W399 ^d^	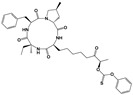	TgHDAC3	DNA expression	11.3 nM/ RH/ HFF	ND	[53]
ELQ-271 ^e^	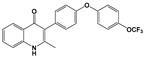	Cytochrome bc1 complex (Qi site)	Cell respiration	0.1 nM/ RH-2F/ HFF Human bc1 inhibition at 800 nM	ND	[54,55,56,63]
ELQ-316 ^e^	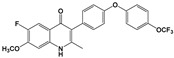	Cytochrome bc1 complex (Qi site)	Cell respiration	0.007 nM/ 2F/ HFF No human bc1 inhibition Not toxic to HFF/HepG2 cells at 10 µM	88% reduction in the number of brain cysts/ ME49/ CBA/J mice/ Intraperitoneal	[54,55,56,63]
ELQ-400 ^e^	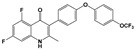	Cytochrome bc1 complex (Qi and Qo site)	Cell respiration	5 µM/ RH/ HFF	Increased mice survival and reduction in brain and spleen parasite load/ RH/ CF-1 mice/ Intraperitoneal	[56,64]
MMV675968 ^f^	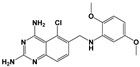	DHFR	Folate synthesis	0.02 µM/ RH/ Vero High selectivity towards parasite DHFR	ND	[65]
Buparvaquone (MMV689480) ^f^	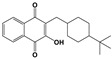	Mitochondrial electron transport chain enzymes (dehydrogenase enzymes)	Cell respiration	0.10 µM/ RH/ Vero	ND	[65]
Tanshinone IIA ^g^	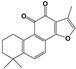	NK	NK	2.5 µM/ PLK/ HFF No host cell toxicity at 25 µM Reduced number of in vitro-induced bradyzoites at 1 µM	ND	[66]
Hydroxyzine ^g^	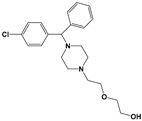	NK	NK	1.0 µM/ PLK ^h^/ HFF No host cell toxicity at 25 µM Reduced number of in vitro-induced bradyzoites at 2 µM	ND	[66]

Abbreviations: ND: not done; NK: not known; IC_50_: half-maximal inhibitory concentration; TgCDPK1: *T. gondii* calcium-dependent protein kinase 1; ENR: enoyl-acetyl carrier protein reductase; KAS I/II: β-ketoacyl-acyl carrier protein synthase; FAS II: type II fatty acid synthesis; TgHDAC3: *T. gondii* histone deacetylase 3; DHFR: dihydrofolate reductase; HFF: human foreskin fibroblasts; IFN: interferon; LLCMK2: rhesus monkey kidney epithelial cells; HepG2: human hepatocarcinoma cells. *: Chemical structures were designed using ChemDraw 17.0 software. a: Bumped-kinase inhibitors derived from BKI-1294. b: Triclosan analogue. c: Acylsulfonamides originally developed for *Mycobacterium tuberculosis.* d: FR235222 derivatives. e: Endochin-like quinolones. f: Repurposed compounds from the pathogen box (initiative of the Medicines for Malaria Venture). g: Repurposed compounds from the compound library provided by the Drug Discovery Initiative (University of Tokyo). h: Type II strain of *T. gondii*, derived from the ME49 strain.

## Data Availability

No new data were created or analyzed in this study. Data sharing is not applicable to this article.
